# Plasmablastic lymphoma: current knowledge and future directions

**DOI:** 10.3389/fimmu.2024.1354604

**Published:** 2024-02-13

**Authors:** Ji-Wei Li, Hong-Ling Peng, Xiao-Yan Zhou, Jing-Jing Wang

**Affiliations:** ^1^ Department of Oncology, The Second Xiangya Hospital, Central South University, Changsha, China; ^2^ Department of Hematology, The Second Xiangya Hospital, Central South University, Changsha, China; ^3^ Department of Pathology, Fudan University Shanghai Cancer Center, Shanghai, China; ^4^ Department of Oncology, Shanghai Medical College, Fudan University, Shanghai, China; ^5^ Institute of Pathology, Fudan University, Shanghai, China

**Keywords:** plasmablastic lymphoma, HIV, molecular profiles, treatment, immunotherapy

## Abstract

Plasmablastic lymphoma (PBL) is an aggressive non-Hodgkin lymphoma associated with HIV infection and immunodeficiency. However, PBL can also be seen immunocompetent individuals in recent studies. PBL was characterized by distinct clinical and pathological features, such as plasmablastic morphology and universal expression of plasma cell markers. The clinicopathologic features were different between HIV-negative and HIV-positive patients. Gene expression analysis identified the unique molecular feature in PBL, including frequent c-*MYC* rearrangement and downregulation of BCR signaling pathway. Despite the recent advances in the treatment of PBL, the prognosis of PBL patients remains dismal. The objectives of this review are to summarize the current knowledge on the epidemiology, molecular profiles, clinical and pathological features, differential diagnosis, treatment strategies, prognostic factors, and potential novel therapeutic approaches in PBL patients.

## 1 Introduction

Plasmablastic lymphoma (PBL) is a rare subtype of diffuse large B-cell lymphoma (DLBCL), with high invasiveness and poor prognosis (1). Pathologically, the tumor cells showed large cell similar to immunoblastic B cells but expressed plasma cell associated antigens (1). In 1997, Delecluse et al. described 16 cases of primary oral DLBCL with special immunophenotype, of which 15 cases were positive for human immunodeficiency virus (HIV), and proposed the diagnosis of PBL for the first time (2). In 2001, PBL was classified as HIV infection associated lymphoma in the classification of lymphoid and hematopoietic system tumors by World Health Organization (WHO) (3). In 2008, the WHO classification of lymphoid and hematopoietic system tumors separated PBL from DLBCL and classified it as acquired immunodeficiency syndrome associated lymphoma (ARL) (4). In 2016, PBL was classified by WHO as an independent subtype of large B-cell lymphoma (5), which was associated with HIV and EB virus infections, or other immunodeficiency states, such as long-term use of immunosuppressants, solid organ transplantation, or age-related immune decline.

The prognosis of PBL was significantly worse than DLBCL, with a median OS of around 12 months (6–8). although multiple new treatment regimens were developed and tried in PBL, the survival outcome remain poor (9–12). In the past 10 years, due to the rarity of this disease, most of the knowledge about it comes from clinical case reports and the etiology, molecular features and prognostic factors of this entity remain largely unknown (6). In this paper, the etiology, pathological features, treatment and prognostic factors of PBL are reviewed.

## 2 Epidemiology and clinical features

DLBCL and Burkitt’s lymphoma (BL) are the most common subtypes of the AIDS-related lymphomas (ARLs), and PBL represents around 11% of ARLs (13, 14). ARLs account for approximately 3% of non-Hodgkin’s lymphoma (15, 16), however, the exact incidence of HIV-positive PBL is still unknown. In the recent years, an increasing number of PBL cases with normal immune function have been reported (6, 17–19). The clinicopathologic features of PBL were significantly different between HIV positive and HIV negative individuals (18, 20). PBL occurred more commonly in adult men, especially in HIV positive patients (13, 21, 22), with a median age of 46 years old in HIV-positive patients (male/female:8/1) and 57 years old in HIV-negative patients (male/female: 1.7-1.9/1) (6, 20). Of the 135 cases of PBL from the LYSA group (20), HIV positive and negative patients accounted for 42% and 58%, respectively. Around one-third of HIV-negative PBL are associated with immunodeficiency such as solid organ transplantation and steroid hormone use (6, 20). A meta-analysis summarized the reported cases of PBL between 1997 and 2015 in China and the results demonstrated that all the patients were HIV negative (23). Recently, our group reported 56 cases of PBL from China and found that most patients were immunocompetent, and HIV infection was not observed (17). The above results showed that the immune status of PBL was significantly different between the eastern and western population. Similar to ARL such as Burkit lymphoma and primary exudative lymphoma (PEL), PBL is also associated with Epstein-Barr virus (EBV) infection, and Epstein-Barr virus-encoded RNA was positive in over half of the PBL patients (6, 20). The association between PBL and human herpes virus 8 (HHV-8) has yet to be elucidated, and HHV-8-related protein expression has been found in only a few cases (6, 19).

In HIV-negative PBL, the most common sites of extra-oral lesions were gastrointestinal tract, lymph nodes and skin, and extra-nodal lesions accounted for 82% (6, 17, 19). However, oral cavity is involved more frequently in HIV positive PBL than that in HIV negative PBL (6, 20). Only a few cases originate in the central nervous system (CNS), paranasal sinus, mediastinum, subcutaneous, lung and testis (6). The distribution of clinical stage is bimodal, with more than 80% of patients present at stage I and stage IV (6). Approximately 33% of HIV-positive PBL patients and 50% of HIV-negative PBL patients have B symptoms (6, 24). It has been reported that the average time from the diagnosis of AIDS to PBL was 5 years, while PBL was the first symptom in 5% of AIDS case (7). In addition, PBL could also be secondary to plasmacytoma, follicular lymphoma, and Richter’s transformation of chronic lymphocytic leukemia (25–28).

## 3 Etiology and molecular features

The etiology and pathogenesis of PBL remain largely unclear. At present, it is believed that PBL originates from activated B cells in the terminal differentiation stage after the germinal center, and may be in the stage of development and transformation of immunoblastic cells into plasma cells (1). These cells have undergone high frequency of somatic mutations and immunoglobulin (lg) class switching. During this process, intracellular molecular signaling pathways and chromosomal abnormalities may lead to malignant transformation. *MYC* gene rearrangement (at 8q24) was the first cytogenetic abnormality identified in PBL patients [3]. *MYC* gene rearrangement was detected in over half of PBL patients (18, 29–32) and Ig gene was the main partner of *MYC* gene rearrangement (29). *MYC* gene rearrangement was more common in EBER positive patients (74%) than in EBER negative patients (43%) (29). In addition, the *MYC* rearrangement rate was significantly higher in EBV-positive PBL patients than that in EBV-negative patients (33). Targeted sequencing showed tha*t MYC* translocations was observed in as high as 87% PBL cases (34). The role of *MYC* gene rearrangement in the pathogenesis of PBL is not clear. It is believed that the plasmablastic morphology of tumor cells and the aggressiveness of PBL are related to MYC gene rearrangement.

Notch1 is an important regulatory signal for T - and B-lineage selection during lymphoid progenitor cell development, and it can inhibit the expression of some transcription factors in B-lineage lymphocytes. Notch l is also involved in signaling pathways associated with cell proliferation and survival, including mammalian target of rapamycin (mTOR) (35). Notch1 pathway was demonstrated to be activated in PBL by whole exome sequencing (WES) (36). Segmiller et al. found that Notch1 was detected by immunohistochemistry (IHC) in all 9 cases of PBL (37). The positive rates of mTOR substrate phosphorylated ribosomal protein S6 (mps6) and eukaryotic initiation factor 4E binding protein 1 (4EBP1) in PBL were 100% and 86%, respectively (37), which were similar to those in 5 PEL cases and 21 plasma cell myeloma cases. Notch protein may inhibit the normal phenotypic expression of B cells and activate mTOR signaling pathway.

Previous studies showed that the gene profiles and mutation spectrum were significantly different between PBL and DLBCL (17, 38). Gene expression analysis has identified the downregulation of B-cell receptor signaling genes in PBL compared to DLBCL (38). In contrast, mitochondrial genes such as ATP5G1, CYC1, NDUFAF1, NDUFB6, NDUFB7 and UQCRQ, were higher in PBLs than DLBCL (38). Our previous study performed RNA-sequencing to identify the molecular features of PBL and the results showed that compared with DLBCL, some biological pathways were significantly downregulated in PBL, including BCR and TCR signaling pathways, whereas many pathways, such as cell adhesion molecules, calcium, and Wnt signaling pathways, were upregulated in PBL (17).

Matsuki et al. (39) first established PBL cell lines *in vitro* by incubating immunodeficient mice subcutaneously with lymph node biopsies from patients with PBL and culturing subcutaneous masses of mice. Comparison of this cell line with the cell lines from the patient’s lymph node *in vitro* by genetic hybridization (CGH) and FISH revealed that t (9: The t (9:13) (p22; q22) and 1(4;7) (q35; q22) chromosomal translocations were observed in the former cell line could cause the loss of tumor suppressor gene p16 and thus upregulated the MDR-1 expression, which is related to the drug resistance.

## 4 Pathological features

Histologically, the tumor cells showed a morphologic spectrum ranging from immunoblastic to plasmacytoid (1). Monomorphic plasmablastic cell morphology was more common in HIV infected patients and was more likely to occur in the mouth, nose and paranasal region. PBL with plasmacytic differentiation was more likely to occur in the extraoral cavity. The “starry sky phenomenon” can be seen, including scattered mature small lymphocytes with frequent mitoses, occasional apoptosis cells and tingible body macrophages (1). However, PBL needs to be distinguished from other large B-cell lymphomas in morphology, Such as plasmablastic plasma cell lymphoma, Burkitt lymphoma, anaplastic lymphoma kinase (ALK) positive anaplastic DLBCL, primary exudative lymphoma (PEL), multicentric Castleman large B-cell lymphoma and HHV-8 positive DLBCL (1). It can be differentiated by clinical history, site of disease, immunophenotype of tumor cells, and EBER detection.

PBL had an immunophenotype of terminally differentiated B cells (6, 17, 20). The markers of mature B cells, such as CD19, CD20, PAX-5, and leukocyte common antigen CD45, and markers of mature T cells, such as CD2, CD3, CD5, and CD7, generally did not express or weakly expressed (6). However, the tumor cells universally expressed markers of plasma cells, such as CD38, Vs38c, CD138 and IRF4/MUM1 (6). Most of the HIV-negative patients had a Ki-67 index higher than 80% (6). Immunohistochemistry showed differences between HIV positive and negative patients, the former had significantly higher CD20 and CD56 expression than the latter (6, 7, 19, 20). The overall positive rate of CD56 was around 40% (6). Although EBER was positive in over half of the PBL cases, latent membrane protein 1 (LMP1) was rarely expressed (24). Positive regulatory proteins (PRDMI/BLIMPI) and activated transcription factor (XBPI) associated with the immunophenotypes of terminally differentiated B lymphocytes are shown in PBL (40).

## 5 Survival outcomes and prognostic factors

Previous case reports and literature review demonstrated that PBL is an aggressive lymphoma with poor prognosis, with a median OS of 14-15 months (5-year survival 31%) in HIV-positive patients and 9 months in HIV-negative patients (6, 7, 19, 24). However, some large multicenter studies in the recent years showed that the survival outcome of PBL seems to be better than previous literature reviews (17, 20, 41–43). In 2018, a French group reported 135 PBL patients from LYSA centers and found that the complete response (CR) rate of 55% and the median overall survival (OS) was 32 months (20), which was much better than previous reports (7, 19). Recently, our previous research retrospectively analyzed 56 cases of PBL from three cancer centers in China and found that the 2-year PFS and OS rates were 59.4% and 65.1%, respectively (17). A multi-institutional retrospective study from America demonstrated the outcomes of patients with limited-stage PBL, with a median follow up of 34 months (1–196), the 3-year PFS and OS of the whole cohort were 72% and 79%, respectively. The above results indicated that the prognosis of PBL was better than that reported in case series, especially in limited stage and HIV negative patients.

According to the previous studies, Age>60 years, Ann Arbor stage III or IV, Eastern Cooperative Oncology Group (ECOC) performance status >2, extraoral primary lesions, immunosuppression, bone marrow infiltration and EBER positive were adverse prognostic factors for HIV negative PBL (6, 20). A recent multi-institutional international retrospective study including 281 PBL patients showed that EBV-negative lymphoma, poor performance status, advanced tumor stage, and bone marrow involvement was associated with inferior OS, while immunosuppression and HIV infection did not influence OS (44).

## 6 Treatment

### 6.1 Chemotherapy

Chemotherapy is the first-line treatment for PBL. The median survival (OS) of patients without chemotherapy was around 3 months (7, 19). The NCCN recommends the use of more intensive chemotherapy regimens, such as CODOX-M/IVAC (cyclophosphamide, vincristine, doxorubicin, and high-dose methotrexate alternated with ifosfamide, etoposide, and high-dose cytarabine), dose-modified EPOCH (etoposide, prednisone, vincristine, cyclocarbonamide, and doxorubicin), or Hyper-CVAD (Cyclophosphamide, vincristine, doxorubicin, and dexamethasone alternated with high-dose methotrexate and cytarabine). However, several studies have demonstrated that no survival benefit was obtained in patients who received intensive chemotherapy (**Table 1**) (6, 8, 18, 20). In a group of 35 patients who received CHOP/CHOP-like chemotherapy and 16 patients who received more intensive chemotherapy, there was no statistically significant difference in survival between the two groups (8). Our group summarized 394 reported HIV-negative PBL, including 124 patients treated with CHOP or CHOP-like chemotherapy and 44 treated with intensive chemotherapy, and no survival difference was found between these two groups (6). Since the tumor cells in PBL showed no expression or little expression of CD20, rituximab is only used in a few patients with CD20 expression (17, 20). Although intensive chemotherapy regimens were recommended by NCCN, most of the reported cases received CHOP/CHOP-like chemotherapy and the treatment efficacy remained controversial and need further investigation. For young patients with good performance status and high-risk factors, intensive chemotherapy might be a better choice.

**Table 1:** The survival difference between CHOP and intensive chemotherapy.

	CHOP or CHOP-like chemotherapy	Intensive chemotherapy	Survival outcome	P value
Tchernonog et al. (20)	70	16	Data not shown	>0.05
Hess BT et al. (41)	11	14	3-year OS 84% vs. 73%	>0.05
Li YJ et al. (6)	124	44	mOS: Not reached vs. 23.0m	0.981
Loghavi et al. (14)	8	16	Data not shown	0.078
Castillo et al. (8)	35	16	Data not shown	>0.05

### 6.2 Proteasome inhibitor-bortezomib

**Figure 1:**
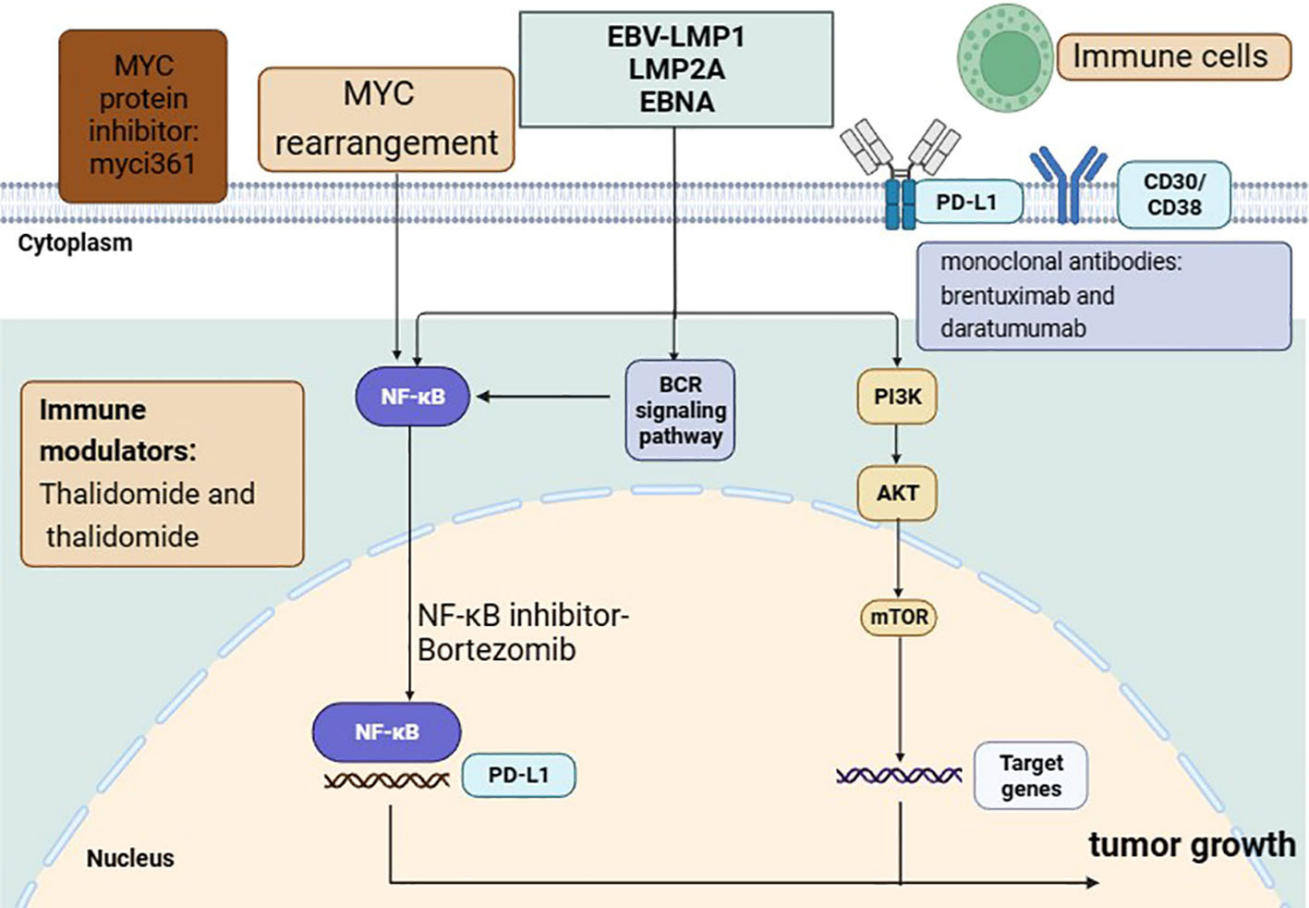
The molecular features and main treatment targets in PBL.

Bortezomib induces apoptosis by blocking the nuclear factor kB (NF-kB) signaling pathway, producing cytotoxic effects in activated B cell type (ABC) DLBCL (**Figure 1**) (45). Bortezomib alone or in combination with chemotherapy (dexamethasone, bortezomib, gemcitabine, Oxaliplatin, cytarabine) may be effective in the treatment of PBL, but the remission was temporary. Bortezomib combined with chemotherapy achieved well results and was tolerated in some PBL patients (**Table 2**). A retrospective study analyzed 8 cases of PBL (5 HIV-positive and 3 HIV-negative) treated with bortezomib combined with EPOCH, producing a CR rate of 100% and 2-year OS rate of 50%, indicating that this regimen was relatively safe and effective for PBL (46). Dittus et al. (47) reported that the CR rate and 2-year OS rate of PBL patients treated with the combination of bortezomib and EPOCH regimen were 100% and 50%, respectively. The 2-year OS rate also exceeded 50% and the ORR was as high as 90% in PBL patients who received bortezomib as a second-line therapy (48). Our previous study reported that the overall response rate of HIV negative patients treated with bortezomib-containing regimens was 71.4%, and the mOS time was only 11 months (17). In summary, bortezomib combined with or without chemotherapy may improve responses and outcomes in PBL, although all studies to date are retrospective and randomized study are still lacking.

**Table 2:** Summary of the efficacy of Bortezomib-based treatment in PBL.

	Number	Treatment response	Survival outcome
Castillo JJ et al. (46)	11	ORR: 100%	Median OS: 11 months
Li YJ et al. (6)	14	ORR: 71.4%	Median OS: 11 months
Dittus C et al. (47)	8	CR:100%	2-year OS: 50% 2-year PFS: 50%

### 6.3 Immune modulators

Thalidomide binds to CRBN targets on tumor cells, promotes ubiquitination and degradation of the transcription factors Ikaros and Aiolos, and activates an interferon-like response, thereby inducing tumor cell apoptosis (49). A newly diagnosed PBL patient achieved CR after fist-line treatment of thalidomide combined with dexamethasone, followed by autologous stem cell transplantation and the patients still maintained CR after 10 years of follow up (50). Lenalidomide is a thalidomide analogue with similar anti-tumor mechanisms. It has been reported that a patient with PBL who progressed after multiple lines of treatment was treated with lenalidomide orally due to severe peripheral neurotoxicity caused by bortezomib, and maintained PR status after 2 years of follow-up (51). Marrero et al. reported that a patient with PBL who relapsed after CHOP regimen was treated with lenalidomide combined with bortezomib as a second-line treatment and still maintained CR status after 12 months of follow-up (11). Although a large number of clinical studies are lacking, lenalidomide alone or in combination with other treatment regimens can help patients maintain long-term CR status for newly diagnosed or relapsed/refractory PBL patients.

### 6.4 Immune checkpoint inhibitors

Programmed death receptor 1(PD-1) expressed by T cells binds to programmed death receptor ligand 1(PD-L1) on the surface of tumor cells, which can inhibit the activation of T cells and induce their apoptosis, leading to the immune escape and tumor progression (52). In PBL, high expression of PD-1 and PD-L1 was detected and the PD-1/PD-L1 pathway was abnormally activated (33, 53–55). Only few reports have demonstrated the efficacy of immune checkpoint inhibitors in PBL patients (10, 56). This patient achieved PR with PD-1 inhibitor monoclonal antibody nivolumab and underwent allogeneic hematopoietic stem cell transplantation without signs of tumor progression as of the time of this article (56). Given the potential activity of PD-1 pathway blockade in PBL, further study of PD-1 blockade is warranted.

### 6.5 CAR-T therapy

Chimeric antigen receptor T cell (CAR-T cell) therapy is a newly developed immunotherapy where T lymphocytes are engineered with synthetic receptors known as chimeric antigen receptors (CAR) (57). The CAR-T cell could produce long-term specific antitumor effects by recognizing and eliminating specific cancer cells. CAR-T cell therapy was an effective anti-tumor for relapsed/Refractory DLBCL (9, 57). Raghunandan et al. reported a case of multiple refractory PBL emerging from B-cell acute lymphoblastic leukemia and failed to allogeneic hematopoietic cell transplant and sustained CR for one year after CAR-T cell therapy (12). Raychaudhuri et al. reported that a patient with PBL who was resistant to traditional chemotherapy, lenalidomide and bortezomib achieved CR after 4 months of CAR-T therapy (Yescarta treatment) (58). As the plasmablastic cells were frequently negative for B cell markers (19, 20), the use of CAR-19 therapies in PBL patients was limited. CAR-T provides a treatment option for patients with relapsed and refractory PBL, but the efficacy needs to be confirmed in the future.

### 6.6 Highly active antiretroviral therapy

HIV patients are often accompanied by CD4+Cell count reduction and immunosuppression (7, 59). The impact of highly active antiretroviral therapy (HAART)on survival outcome in patients with HIV-related PBL remains controversial as the condition is rare and the reported case series is small (7, 19, 60). A retrospective study in the United States explored the effect of HIV on lymphoma and found that HIV was associated with increased risk of death among lymphoma patients in the HAART era (61). Case report showed that a HIV-positive PBL patient achieved sustained remission after HAART alone (60). For HIV-positive patients with PBL, meta-analysis has shown that the combination of highly active antiretroviral therapy (HAART) and chemotherapy and/or radiotherapy can improve the prognosis (7). The possible explanation is that HAART can restore the immune surveillance function of patients so as to play a more effective role in tumor control. However, the prognosis of PBL in HIV-infected individuals remains dismal in the highly active antiretroviral therapy era and intensive chemotherapy regimens did not increase the survival outcome (62).

### 6.7 Hematopoietic stem cell transplantation

Some recent reports have demonstrated the application of autologous hematopoietic stem cell transplantation (ASCT) in PBL patients (**Table 3**) (20, 63, 64). Cattaneo et al. reported 24 PBL patients who received autologous hematopoietic stem cell transplantation and the 2-year OS was 58% (63). A retrospective study of 9 HIV-negative PBL patients from Moffitt Cancer Center showed that four patients received ASCT as consolidation therapy after first complete remission and the survival time was 36.5 months (65). LYSA group retrospectively analyzed 135 cases of PBL, including 6 patients who received autologous HSCT after the first CR, and the result showed that 3 patients remained remission at the last follow-up (13, 17 and 29 months after HSCT), 2 patients relapsed at 8 and 26 months, and 1 died after 78 months of remission (20). Recently, a multi-institutional retrospective study reported 8 cases who underwent Auto-SCT consolidation after chemotherapy and the 3-year PFS and 3-year OS were both 63.0% (41). As the above results were achieved based on the small case series, the clinical efficacy of ASCT in PBL need further investigation.

**Table 3:** A brief summary of autologous hematopoietic stem cell transplantation (ASCT) in PBL.

	n	Survival outcome
Hess BT. et al. (41)	8	3-year PFS: 63%, 3-year OS: 63%
Tchernonog et al. (20)	6	PFS: 8, 13, 17,26, 29, 78
Cattaneo et al. (63)	24	2-year OS: 58%
Hubel K, et al. (64)	24	2-year PFS: 52%, 2-year OS: 70%

### 6.8 Other

Some PBL cells express CD30 on their surface. So far, three patients with relapsed/refractory PBL have been reported to have been treated with CD30 monoclonal antibody brentuximab (66–68). Two patients had significant tumor shrinkage after a few days of treatment with brentuximab, but one of these patients developed multiple mediastinal fistulas due to rapid tumor regression. As PBL showed a plasma cell immunophenotype, CD38 is commonly expressed in PBL (6, 20), and daratumumab can induce NK cells to produce antigen-dependent cell-mediated cytotoxicity (69, 70), suggesting that CD38 monoclonal antibody can be used for the treatment of PBL. Fedele et al. (71) revealed that immunomodulators can lead to Ikaros deletion and then upregulated CD38 expression on the surface of tumor cells, providing a theoretical basis for the combination of anti-CD38 monoclonal antibody and immunomodulators in PBL. Shi et al. (72) found that SLAMF7(CD319/CS1) was detected in PBL, suggesting that it may serve as a potential diagnostic marker and therapeutic target for PBL. MYC rearrangement was observed in around half of the patients and this abnormality could inhibit transcription factor BLIMP-1 and thus promote tumor cell proliferation (73). Han et al. developed a new MYC protein inhibitor (myci361), which could inhibit tumor proliferation and increased the infiltration of the lymphocytes (74), but this drug was in the preclinical stage.

### 6.9 Radiation therapy in limited stage PBL

An increasing number of evidences have suggested that the prognosis of limited-stage PBL was much better than advanced stage patients (20, 41). However, the treatment recommendation of limited-stage was similar to advanced stage patients and many patients with limited-stage disease are treated with aggressive chemotherapy or auto-SCT (6, 7, 19). Previous studies have shown that patients treated with aggressive chemotherapy or consolidation with Auto-SCT had a trend toward better outcomes (63, 75). A recent study demonstrated that limited-stage PBL did not benefit from aggressive frontline treatment, including Hyper-CVAD or auto-SCT consolidation (41). However, improved PFS was observed in patients receiving EPOCH based frontline therapy versus CHOP (HR: 0.23; p<0.05). Patients receiving frontline chemotherapy followed by radiation consolidation had better OS than chemotherapy alone (41).

## 7 Conclusion

PBL is a special type of DLBCL, which often occurs in HIV positive patients, shows immunoblastic morphology but expresses plasma cell markers. Compared with DLBCL, NOS, some important biological pathways were abnormally activated or inactivated in PBL, such as BCR signaling and CAM signaling. As we have mentioned above, the prognosis of PBL was still dismal with current treatment strategies. Although intensive chemotherapy strategy was recommended by NCCN guideline, CHOP or CHOP-like chemotherapy achieved similar efficacy. Chemotherapy followed by radiation consolidation improved the survival outcome of limited-stage PBL and may be potential standard treatment for this group of patients in the future. Bortezomib combined with or without chemotherapy may improve the survival outcomes in PBL, but all studies to date are retrospective and large randomized study are sparse. PD-1/PD-L1 pathway was abnormally activated in PBL, although the efficacy of PD-1 inhibitor was only reported in case report, it may be a promising treatment and need further investigation. Other potential therapeutic approaches for patients include EBV-targeted therapies, including antiviral agents or EBV-targeted cellular immunotherapy, but the efficacy and tolerance of these approaches have not yet been evaluated in PBL patients. New treatment strategies such as thalidomide and anti-CD30 antibodies were explored in case reports, but the exact efficacy of these treatment remain to be validated in the future. It is urgent to further investigate the biological characteristics and develop more effective targeted therapeutic agents for PBL patients.

## Author contributions

J-WL: Investigation, Writing – original draft. H-LP: Supervision, Writing – review & editing. X-YZ: Supervision, Validation, Funding acquisition, Writing – review & editing. J-JW: Supervision, Visualization, Writing – review & editing.
